# Application of the mixture item response theory model to the Self-Administered Food Security Survey Module for Children

**DOI:** 10.1371/journal.pone.0228099

**Published:** 2020-01-23

**Authors:** Isabel Maia, Milton Severo, Ana Cristina Santos

**Affiliations:** 1 EPIUnit—Instituto de Saúde Pública, Universidade do Porto, Rua das Taipas, Porto, Portugal; 2 Departamento de Ciências da Saúde Pública e Forenses e Educação Médica, Faculdade de Medicina, Universidade do Porto, Alameda Prof. Hernâni Monteiro, Porto, Portugal; Hong Kong Polytechnic University, HONG KONG

## Abstract

**Background:**

The Self-Administered Food Security Survey Module for Children was developed to assess food insecurity of individual children and has not been used in Portugal. We aimed to apply the mixture item response theory model to the Self-Administered Food Security Survey Module for Children, to assess its reliability and validity, and to estimate the cut-offs of the food security status for Portuguese children.

**Methods:**

The scale was self-administered to 2132 children of the Generation XXI birth cohort. The internal consistency was assessed using Cronbach’s alpha. We evaluated dimensionality and/or clustering, and Latent Class Analysis, Latent Trait Analysis and Mixture Latent Trait Analysis were tested. The number of classes and/or traits were defined according to the Akaike Information Criterion, Bayesian Information Criterion, Adjusted Bayesian Information Criterion, Vuong-Lo-Mendell-Rubin Likelihood Ratio Test, Bootstrapped Likelihood Ratio Test and Entropy. Construct validity was explored using socio-demographic characteristics. The classification tree was used to define cut-offs to predict cluster membership.

**Results:**

The best model was a Mixture Latent Trait Analysis with 1 factor and 2 classes (food security and food insecurity), assuming class variant item parameters (for items 1 and 3). Based on the estimated posterior probabilities, the food insecurity prevalence was 17.6%. Cronbach’s alpha was 0.617. A higher proportion of less-educated mothers and low-income households was observed in the food insecurity class. The classification tree showed an accuracy of 100.0% by identifying the food security and food insecurity groups.

**Conclusion:**

Our results supported that the Self-Administered Food Security Survey Module for Children provides a valid and reliable measure, which allows the identification of food insecurity among Portuguese children.

## Introduction

Food insecurity, defined as “limited or uncertain availability of nutritionally adequate and safe foods or limited or uncertain ability to acquire acceptable foods in socially acceptable ways” [1], is widely considered a public health concern, and the magnitude of this problem is even more dramatic when is experienced by children. There is increasing evidence in the literature on the negative health outcomes associated with food insecurity, particularly among children [2–7], reinforcing the relevance of its assessment at this period of life.

The growing interest in food insecurity led to the development of tools that allow the assessment of food security status. Among these tools, the United States Household Food Security Survey Modules (HFSSMs) have been widely used, being subjected to cultural adaptations and used in different socioeconomic and cultural contexts [8]. Although the 18-item HFSSM assesses the dimension of food insecurity in children [9], it is mainly a household’s measure [10], often reported by the mother or another adult of the household, not using the children’s individual perception. Moreover, the degree of food insecurity and its perception can differ among individuals within the household, and especially, children can experience food insecurity differently than adults [11], supporting the need to assess children’ perceptions [12].

A food security survey module to be administered to children was developed by researchers at the University of Southern Mississippi in collaboration with Economic Research Service of the United States Department of Agriculture, and documented in 2004 by Connell and colleagues [13]. This tool revealed to provide reliable measures of food insecurity among children [13], being self-administered, composed of nine items, and children’s food security status defined based on the number of affirmative responses [14].

Food insecurity is considered a latent variable, this is, a variable that is not directly observable, but that can be estimated through answers to a set of dichotomous items [15]. To model this type of data with observed items and latent variables, the most usual approaches include latent class analysis (LCA), latent trait analysis (LTA) and mixture latent trait analysis (MLTA) [16].

Briefly, LCA groups individuals into classes of latent variable—food insecurity—based on the responses to the scale items. On the other hand, LTA, also known as item response theory, set individuals and items on the same continuum, providing a continuous score of the latent variable that accounts for the differences between individuals. Finally, the MLTA represents the mixture of the LCA and LTA, allowing the classification of individuals into groups but, at the same time, accounting for the differences within groups [16–18].

To the best of our knowledge, the Self-Administered Food Security Survey Module for Children has not been used in Portugal, which reinforces the need to evaluate its validity when assessing food security status among Portuguese children.

Therefore, we aimed to assess the reliability and validity of the Self-Administered Food Security Survey Module for Children among Portuguese population and to estimate cut-offs of food security status among Portuguese children from the population-based birth cohort Generation XXI.

## Methods

### Participants

The present study was based on data from the Portuguese population-based birth cohort Generation XXI, as described elsewhere [19, 20]. From April 2005 to August 2006, 8647 live newborns born in the five public maternity units of Porto Metropolitan Area, Portugal, were enrolled. Follow-up evaluations took place at 4, 7 and 10 years of age.

The 10-year-old follow-up started in July 2015 and ended on July 2017, and 6397 children were assessed. Of those, in a sub-sample of 2209 children food security status was assessed.

For the present study, singletons or one of the children in the case of multiple births were considered (n = 2156). Participants that reported read (n = 4) or cognitive difficulties (n = 2) or a sibling (n = 1) were not included. Those with missing information on at least one item of the scale were removed from the analysis (n = 17), leading to a final sample of 2132 children.

This study was conducted according to the Ethical Principles for Medical Research Involving Human Subjects laid down in the Declaration of Helsinki and all procedures were approved by the Ethics Committee of University of Porto Medical School/ Centro Hospitalar São João. Generation XXI was also approved by the Portuguese Data Protection Authority. Written informed consent was obtained from all the parents or legal representatives in the recruitment and the subsequent follow-up evaluations.

### Data collection

Data were collected using structured questionnaires. Food security status was assessed using the Self-Administered Food Security Survey Module for Children [14]. The scale was self-administered to children, who answered separately from the parents or accompanying adult(s), to guarantee confidentiality and to avoid any constraint.

Socio-demographic data were reported by parents or children’s caregiver. Data on caregivers’ unemployment (if none, at least one or both caregivers had been unemployed at least once a time since 2009), average household monthly income, household income perception, household size and the type of school attended by the child were collected at the 10-year-old follow-up evaluation. In addition, information on maternal education reported at baseline and the number of homerooms reported at 7 years of age were also used.

#### Self-Administered Food Security Survey Module for Children

The scale was composed of nine items with three answer options (“a lot”, “sometimes” and “never”) about the food situation in the household, related to the month previous the evaluation [14]. Items were related to worrying that food at home would run out before the family got money to buy more, food running out, meals including only a few kinds of cheap food, not able to eat a balanced meal, eating less, cutting the size of meals, skipping a meal, being hungry and did not eat, and not eating for a whole day, because the children’s family had not enough money for food. For each item, answers “a lot” and “sometimes” were considered affirmative, while “never” was considered a negative response. The raw score of the scale corresponded to the sum of affirmative responses in all of the nine items of the scale, which ranged between zero and nine.

The Self-Administered Food Security Survey Module for Children [14], originally written in English, was translated to Portuguese by two researchers with expertise in nutrition sciences and epidemiology. In item 4 (*How often were you not able to eat a balanced meal because your family didn’t have enough money*?), the term “balanced meal” was substituted by “healthy meal” (“refeição saudável”, in Portuguese) due to the need of cultural adaptation. The remaining structure was kept. The order of the scale items was the same as in the USDA’s scale version [14]. The scale was back-translated into English by an independent professional translator and concomitantly native English speaker (blinded from the original version). The two versions were compared and did not present significant differences.

### Statistical analysis

The internal consistency of the scale, as an indirect measure of reliability, was assessed using Cronbach’s alpha coefficient.

We tested different latent variables models, in order to find the model that best fits the data. The modelling framework used was the following: first, we modelled data using LCA and established the best number of classes; secondly, we modelled data using a Rasch LTA and established the best number of traits/factors; finally, we modelled data using MLTA to find the best combination of both (trait and classes) (S1 Fig). In MLTA, we tested six MLTA models with 1 factor with 2 classes. In model 1, we assumed invariant item parameters (Figure C in S1 Fig). The unstandardized factor loadings were estimated to be equal in all items within and between classes. The thresholds were estimated to be equal between classes. The factor mean and variance were different across classes, with factor mean and variance equal to 0 and 1 in class 2, and freely estimated for class 1; considering this, the standardized factor loadings were unequal between classes. In model 2, the unstandardized factor loadings were estimated to be equal in all items within and between classes. The thresholds were estimated to be unequal between classes for two items (item 1 and item 3, related to worrying that food at home would run out before family got money to buy more, and meals only including a few kinds of cheap foods because the family was running out of money to buy food, respectively), but equal for the remaining items. We performed a sensitivity analysis, where we checked, for each item, if the threshold was similar or different between classes. We observed that only the items 1 and 3 showed different thresholds. The factor mean and the variance was fixed at 0 and 1 for class 2, and freely estimated for class 1; the standardized factor loadings were unequal between classes. In model 3, the unstandardized factor loadings were estimated to be equal in all items within and between classes. The thresholds were estimated to be different between classes, except for item 9 (related to not eating for a whole day because the family did not have enough money for food). The factor mean and the variance was fixed at 0 and 1 for both classes. The standardized factor loadings were equal between classes. In model 4, the unstandardized factor loadings were equal within and between classes. The thresholds were estimated to be different between classes, except for item 9. The factor mean was fixed at 0 for both classes, and the variance was estimated to be equal to 1 in class 2, and freely estimated for class 1; the standardized factor loadings were different between classes. In model 5, the unstandardized factor loadings were equal within and between classes. The thresholds were estimated to be different between classes. The factor mean was fixed at 0 for both classes, and the variance was estimated to be different between classes; the standardized factor loadings were different between classes. In model 6, the unstandardized factor loadings were estimated to be different within and between classes. The thresholds (except for the item 9) were estimated to be different for each item and class. The factor mean and variance were fixed at 0 and 1 for both classes; the standardized factor loadings were different within and between classes. Finally, we also tested a MLTA model with 1 factor and 3 classes.

The maximum likelihood estimator with robust standard errors using a numerical integration algorithm was used. The number of random starts for each model was 90.

Model fitting was assessed by comparing the observed frequency of each response pattern with the expected frequency estimated by each model. The number of classes and/or traits were defined according to the Akaike Information Criterion (AIC), Bayesian Information Criterion (BIC), Adjusted Bayesian Information Criterion (ABIC), Vuong-Lo-Mendell-Rubin Likelihood Ratio Test (VLMR), Bootstrapped Likelihood Ratio Test (BLRT) and Entropy [21–27]. Lower values of AIC, BIC and ABIC indicated better model fit. The VLMR and BLRT compared a k classes with k-1 classes models with the same parameterization. A *p*-value lower than 0.05 indicated that the k model is better. For entropy, the nearest to one, the better the distinction between the classes. AIC, BIC, ABIC and Entropy were used to compare models that are not nested, while the VLMR and BLRT were used to compare nested models.

The MLTA was described according to the item operation characteristic curves of an item that is characterized by two parameters: the unstandardized factor loading (discrimination), which is the same in all the items for all classes, and the thresholds (difficulty), that are as many as the number of categories minus one. In this specific case, as we have two categories in each item, we have one threshold for each item. Each item operation characteristic curve represents the probability of endorsing affirmatively the item according to the latent trait variable value; the threshold value corresponds to the latent trait variable value at which the probability of endorsing affirmatively an item is 0.5.

Differential Item Functioning (DIF) was tested. An item was considered to have a DIF when the logistic regression showed a significant association between classes and the probability of endorsing affirmatively an item, after adjusting for the factor score. The classes and factor scores used in the logistic regression was the model of invariant item parameters (model 1).

The prevalence of food insecurity was estimated based on the estimated posterior probabilities.

All individuals were classified according to the most likely class membership.

To explore the construct validity of the Self-Administered Food Security Survey Module for Children, maternal education, type of school, number of homerooms, household income perception, average household monthly income, household size, household crowding (defined as more than 1.5 persons per room [28, 29]) and caregivers’ unemployment were compared across the two classes of food security status.

Categorical variables were summarized as absolute and relative frequencies and compared using the Chi-square test. Continuous variables were described as mean and standard deviation (SD). Normal distributed continuous variables were compared using student’s *t*-test.

A classification tree was used to identify the cut-offs to predict the clusters membership that better distinguishing between the two classes [30]. For that purpose, the raw score of the scale and each item separately were included. Using the classification tree, a simple algorithm to classify the individuals in the classes is obtained.

Considering the second objective, we measure the accuracy between the classification tree and the class membership from the MLTA.

A significance level of 0.05 was used. Mplus version 5.2 and SPSS statistics 25.0 were used. To obtain the classification tree, rpart was used [31].

## Results

At the time of food security status evaluation, the children included in the present analysis had a mean (SD) age of 11.0 (0.23) years and 47.9% of children were girls.

The highest proportion of affirmative responses (53.0%) was observed for the first scale item, related to worrying that food at home would run out before family got money to buy more, whereas the ninth item, related to not eating for a whole day because the family did not have enough money for food, presented the lowest proportion of affirmative response (0.4%).

The tested fit parameters established a latent class model with 3 classes as the optimum number of classes in the LCA (Table 1 and S1 Appendix). In the LTA, the 1 trait/factor model was the optimum number of factors, as the exploratory factor analysis showed that the eigenvalue of the second factor was lower than 1 [32], meaning that the second factor explained a small part of the variance.

**Table 1:** Information criteria for each model parameterization of the food security data.

	Number of Free Parameters	AIC	BIC	ABIC	VLMR (*p*-value for k-1)	BLRT (*p*-value for k-1)	Entropy
**LCA**							
LCA 1 class	9	11153	11204	11176	-	-	-
LCA 2 classes	19	10099	10207	10146	<0.001	<0.001	0.761
LCA 3 classes	29	10021	10185	10093	<0.001	<0.001	0.668
LCA 4 classes	39	10025	10246	10122	0.354	0.650	0.668
**LTA**							
1-f*	10	10072	10129	10097	-	-	-
**MLTA**							
MLTA 1-f 2 class^†^ (Model 1)	12	10071	10139	10100	0.062	0.067	0.211
MLTA 1-f 2 classes^‡^ (Model 2)	14	**10030**	**10109**	**10064**	**<0.001**	**<0.001**	**0.559**
MLTA 1-f 2 classes^§^ (Model 3)	18	10033	10135	10078	<0.001	<0.001	0.514
MLTA 1-f 2 classes^ǁ^ (Model 4)	19	10029	10137	10076	0.020	<0.001	0.602
MLTA 1-f 2 classes^¶^ (Model 5)	21	10018	10137	10070	<0.001	0.025	0.636
MLTA 1-f 2 classes** (Model 6)	36	10010	10214	10099	0.594	0.292	0.772
MLTA 1-f 3 classes	19	10032	10140	10080	0.565	0.373	0.665

ABIC, Adjusted Bayesian Information Criterion; AIC, Akaike Information Criterion; BIC, Bayesian Information Criterion; BLRT, Bootstrapped Likelihood Ratio Test; f, Factor; LCA, Latent Class Analysis; LTA, Latent Trait Analysis; MLTA, Mixture Latent Trait Analysis; VLMR, Vuong-Lo-Mendell-Rubin Likelihood Ratio Test

* The same model as the MLTA 1 factor 1 class

^†^ The unstandardized factor loadings were estimated to be equal in all items within and between classes. The thresholds were estimated to be equal between classes. The factor mean and variance were different across classes, with factor mean and variance equal to 0 and 1 in class 2, and freely estimated for class 1; considering this, the standardized factor loadings were unequal between classes

^‡^ The unstandardized factor loadings were estimated to be equal in all items within and between classes. The thresholds were estimated to be unequal between classes for two items (item 1 and item 3), but equal for the remaining items. The factor mean and the variance was fixed at 0 and 1 for class 2, and freely estimated for class 1; the standardized factor loadings were unequal between classes

^§^ The unstandardized factor loadings were estimated to be equal in all items within and between classes. The thresholds were estimated to be different between classes, except for item 9 (related to not eating for a whole day because the family did not have enough money for food). The factor mean and the variance was fixed at 0 and 1 for both classes. The standardized factor loadings were equal between classes

^ǁ^ The unstandardized factor loadings were equal within and between classes. The thresholds were estimated to be different between classes, except for item 9. The factor mean was fixed at 0 for both classes, and the variance was estimated to be equal to 1 in class 2, and freely estimated for class 1; the standardized factor loadings were different between classes

^¶^ The unstandardized factor loadings were equal within and between classes. The thresholds were estimated to be different between classes. The factor mean was fixed at 0 for both classes, and the variance was estimated to be different between classes; the standardized factor loadings were different between classes

** The unstandardized factor loadings were estimated to be different within and between classes. The thresholds (except for the item 9) were estimated to be different for each item and class. The factor mean and variance were fixed at 0 and 1 for both classes; the standardized factor loadings were different within and between classes.

According to the majority of the fit parameters tested, the best model showed to be the MLTA—1 factor and 2 classes model (Model 2) (Table 1 and Figure D in S1 Fig). Compared to model 1, on which the thresholds were invariant, model 2, with unequal thresholds between classes for the item 1 and item 3, but equal for the remaining items, showed to be better.

The two identified classes–class 1 and class 2 –were defined as food security and food insecurity, respectively.

The average latent class probabilities for most likely latent class membership was 0.89 for food security class and 0.79 for food insecurity class.

Based on the estimated posterior probabilities, the prevalence of food insecurity in the studied sample of children was 17.6%.

**Fig 1:**
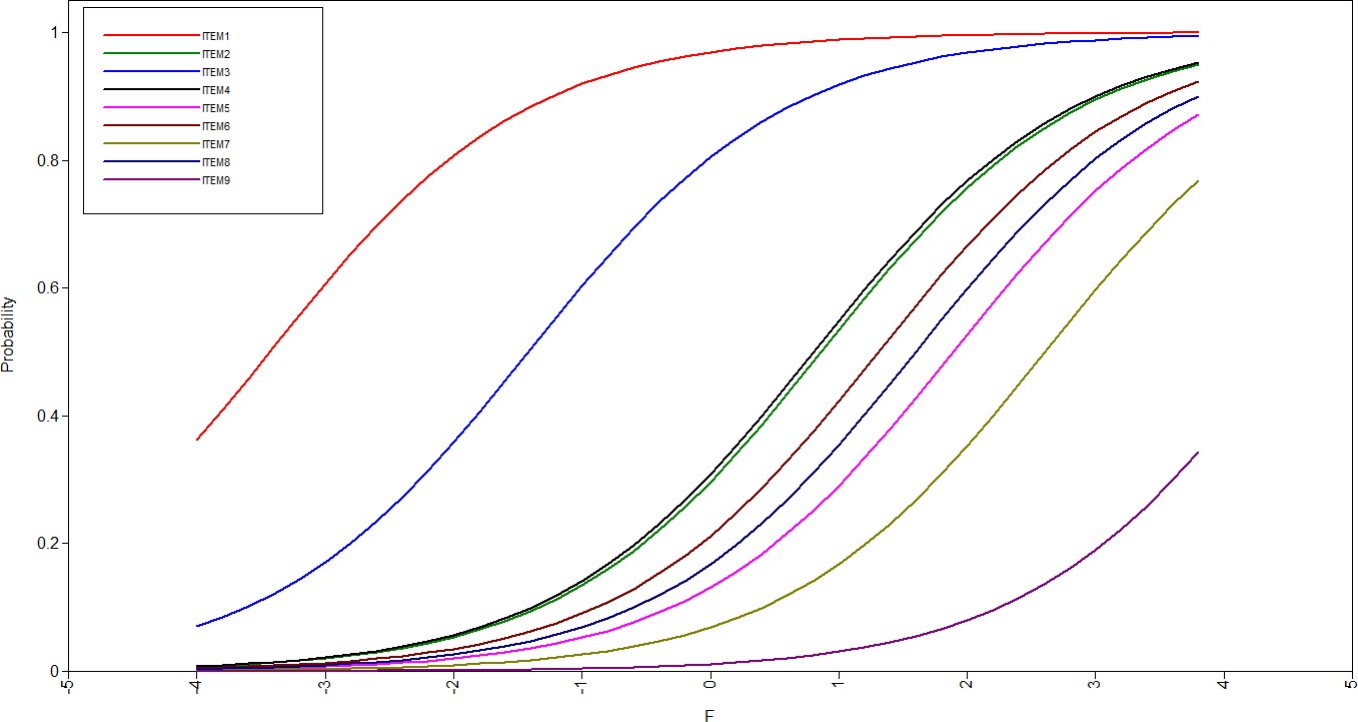
Item operation characteristic curves in the food security class for the nine items of the Self-Administered Food Security Survey Module for Children. F represents the latent trait variable.

**Fig 2:**
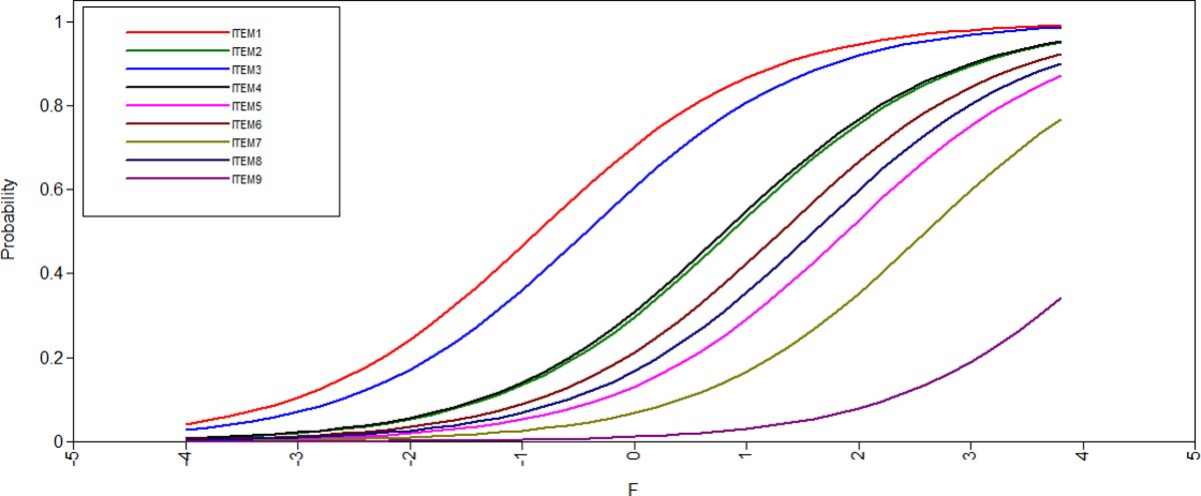
Item operation characteristic curves in the food insecurity class for the nine items of the Self-Administered Food Security Survey Module for Children. F represents the latent trait variable.

The mean in class 1 (food security class) were lower than in class 2 (food insecurity class), representing that, in this class, it was less likely to provide affirmative responses than in the food insecurity class. Even that, slightly lower thresholds for the items 1 and 3 were observed in the food security class compared to the food insecurity class (Table 2, Fig 1 and Fig 2). The food security class (standardized factor loading (standard error) = 0.646 (0.015)) discriminated better the individuals than the food insecurity class (standardized factor loading (standard error) = 0.483 (-)).

**Table 2:** The proportion of affirmative responses for each item of the Self-Administered Food Security Survey Module for Children and corresponded thresholds according to the classes of food security status.

	Proportion of Affirmative Responses (%)	Unstandardized Thresholds			
		Class 1—Food Security (82.4%)*		Class 2—Food Insecurity (17.6%)*	
Items		T (s.e.)	*p*	T (s.e.)	*p*
Item 1—*Did you worry that food at home would run out before your family got money to buy more*?	53.0	-3.433 (0.202)	<0.001	-0.862 (0.297)	0.004
Item 2—*Did the food that your family bought run out*, *and you didn’t have money to get more*?	8.7	0.864 (0.322)	0.007	0.864 (0.322)	0.007
Item 3—*Did your meals only include a few kinds of cheap foods because your family was running out of money to buy food*?	26.0	-1.417 (0.248)	<0.001	-0.427 (0.227)	0.060
Item 4—*How often were you not able to eat a balanced meal because your family didn’t have enough money*?	9.0	0.807 (0.319)	0.011	0.807 (0.319)	0.011
Item 5—*Did you have to eat less because your family didn’t have enough money to buy food*?	4.1	1.892 (0.322)	<0.001	1.892 (0.322)	<0.001
Item 6—*Has the size of your meals been cut because your family didn’t have enough money for food*?	6.4	1.311 (0.310)	<0.001	1.311 (0.310)	<0.001
Item 7—*Did you have to skip a meal because your family didn’t have enough money for food*?	2.3	2.607 (0.331)	<0.001	2.607 (0.331)	<0.001
Item 8—*Were you hungry but didn’t eat because your family didn’t have enough food*?	5.2	1.601 (0.315)	<0.001	1.601 (0.315)	<0.001
Item 9—*Did you not eat for a whole day because your family didn’t have enough money for food*?	0.4	4.452 (0.435)	<0.001	4.452 (0.435)	<0.001
**Unstandardized Factor Loading**		1.000 (-)	-	1.000 (-)	-
**Standardized Factor Loading**		0.646 (0.015)		0.483 (-)	
**Mean (s.e.)**		-3.435 (0.192)		0 (-)	
**Variance (s.e.)**		2.352 (0.569)		1 (-)	

*p*, p-value; s.e., standard error; T, threshold value

* Based on the estimated posterior probabilities

The Self-Administered Food Security Survey Module for Children showed to have an acceptable internal consistency (Cronbach’s alpha = 0.617).

### Construct validity

Considering previously established hypothesis from the literature, on which low parental education [3, 33], unemployment [33–35], larger households [36, 37] and insufficient household income [3, 33, 38] were characteristics associated with food insecurity, the construct validity was explored. The number of homerooms, household crowding and the type of school attended by children were also used.

Children from food insecurity class had less educated mothers and their families reported more often a low and insufficient household income, as well as to be composed of more persons. Moreover, children classified in the food insecurity class had more frequently caregivers that had been at least once unemployed since 2009 and had a higher number of persons per room. Also, children who belong to the food insecurity class more often attended a public school than children in the food security class (Table 3).

**Table 3:** Socio-demographic characteristics of the Generation XXI children according to food security classes.

	Total (n = 2132)	Food Security [1919 (90.0)]*^†^	Food Insecurity [213 (10.0)]*^†^	*p*
**Maternal education** (years) (mean (SD))	2122 (99.5) 11.5 (4.3)	11.7 (4.2)	10.0 (4.3)	<0.001
Type of school^*^	2131 (100.0)			0.050
Private	235 (11.0)	220 (11.5)	15 (7.0)	
Public	1896 (89.0)	1698 (88.5)	198 (93.0)	
Number of homerooms^*^	2130 (99.9)			0.178
≤3	748 (35.1)	665 (34.7)	83 (39.0)	
4	834 (39.2)	748 (39.0)	86 (40.4)	
≥5	548 (25.7)	504 (26.3)	44 (20.6)	
Household income perception^*^	2101 (98.5)			
Insufficient	80 (3.8)	61 (3.2)	19 (9.0)	<0.001
Need to be careful	498 (23.7)	429 (22.7)	69 (32.5)	
Enough to meet needs	958 (45.6)	868 (46.0)	90 (42.4)	
Comfortable	565 (26.9)	531 (28.1)	34 (16.0)	
**Average household monthly income** (€)^*^	2079 (97.5)			<0.001
≤1000	491 (23.6)	410 (21.9)	81 (39.7)	
1001–1500	617 (29.7)	557 (29.7)	60 (29.4)	
1501–2000	406 (19.5)	376 (20.0)	30 (14.7)	
2001–2500	274 (13.2)	258 (13.8)	16 (7.8)	
>2500	291 (14.0)	274 (14.6)	17 (8.3)	
Household size^*^	2130 (99.9)			<0.001
2 persons	80 (3.8)	66 (3.4)	14 (6.6)	
3 persons	631 (29.6)	579 (30.2)	52 (24.5)	
4 persons	1051 (49.3)	961 (50.1)	90 (42.4)	
≥5 persons	368 (17.3)	312 (16.3)	56 (26.4)	
Household crowding^*^	2128 (99.8)			0.030
≤1.5	2038 (95.8)	1841 (96.1)	197 (92.9)	
>1.5	90 (4.2)	75 (3.9)	15 (7.1)	
Caregivers unemployment^*^	2129 (99.9)			0.001
No	1094 (51.4)	1011 (52.7)	83 (39.2)	
At least one caregiver	841 (39.5)	739 (38.5)	102 (48.1)	
Both caregivers	194 (9.1)	167 (8.7)	27 (12.7)	

*p*, *p*-value; SD, standard deviation

* n (%)

^†^ Based on the most likely class membership

### Identification of food security status clusters

**Fig 3:**
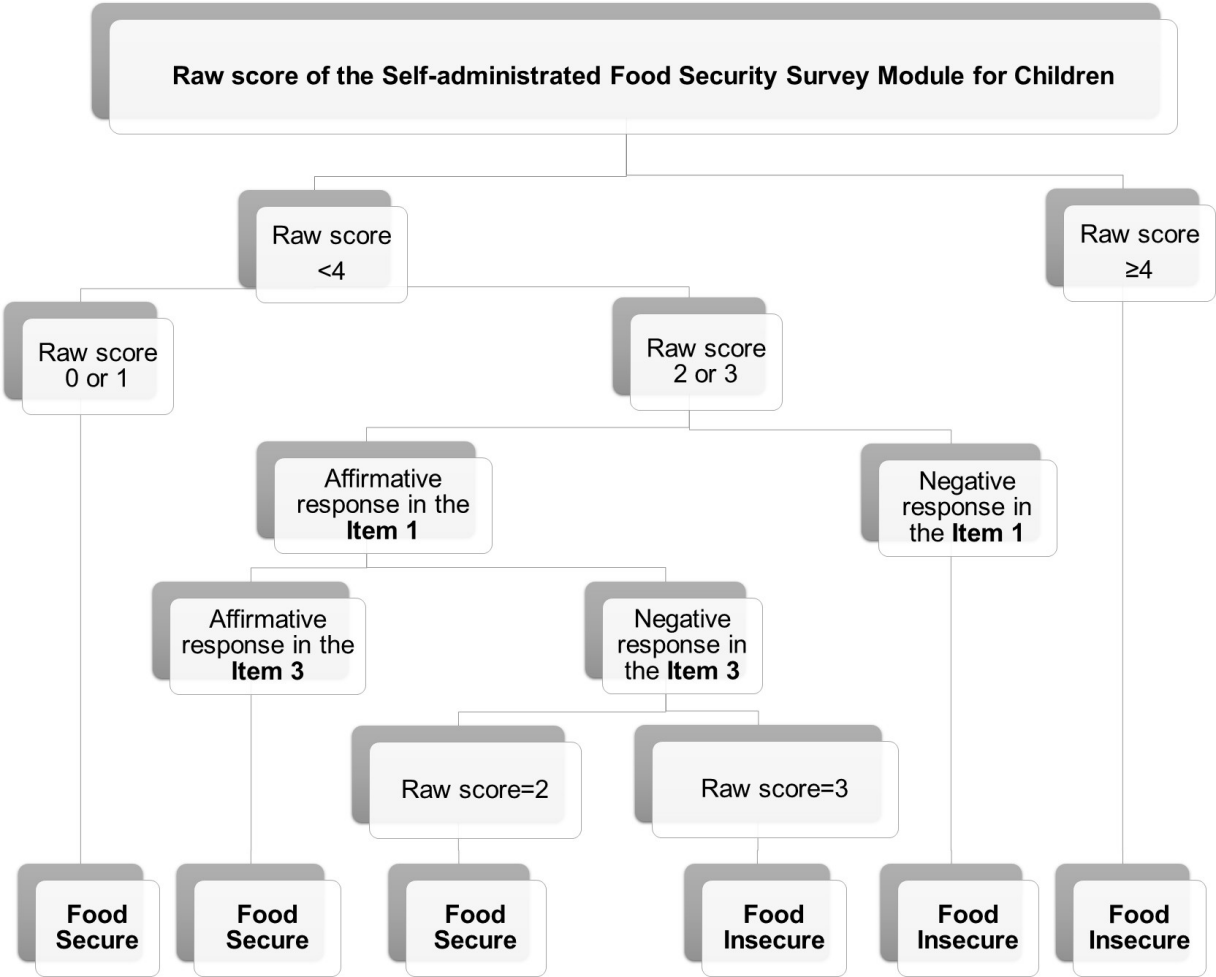
Classification tree, showing the cut-off points for the food security status classes.

From the classification tree, the individuals can be classified as food insecure, if the raw score was equal to or higher than four, if the raw score was equal to two or three, including a negative response in item 1 (worrying that food at home would run out), and if the raw score was equal to three, including an affirmative response to the item 1 and a negative response in item 3 (meals only including a few kinds of cheap foods). Individuals can be classified as food secure if the raw score was lower than or equal to one, if the raw score ranged between two and three, including affirmative responses for the item 1 and 3, and if the raw score was equal to two, including an affirmative response in item 1, but a negative response in the item 3 (Fig 3).

High accuracy was verified (100%) between the observed class identified through MLTA and the classification tree.

## Discussion

In this study, we aimed to assess the reliability and validity of the Self-Administered Food Security Survey Module for Children, as well as to estimate the categories and respective cut-offs of food security status.

According to our results, the Portuguese version of the Self-Administered Food Security Survey Module for Children has a Cronbach’s alpha of 0.617. As, the Cronbach’s alpha is higher than 0.6, it represents an acceptable internal consistency [39, 40]. Also, this value is similar to that reported for the original scale [13], further supporting our findings.

Among the models performed to estimate the food security status classes, the best solution was found for the MLTA (1 factor and 2 classes, class variant item parameters (model 2)). This model showed that the expected values for the response pattern were close to the observed values (S2 Appendix). Using the MLTA approach, we identified two classes, with a high degree of separation, allowing the classification of children as being food secure and food insecure.

In fact, recently, it was reported the use of a similar approach in the Brazilian population, using the Brazilian Household Food Insecurity Measurement Scale [41, 42], and one of the studies encouraged the application of this methodology to other contexts [41].

Using the current classification guidelines of the Self-Administered Food Security Survey Module for Children, four classes of food security status (high food security, marginal food security, low food security, and very low food security) can be identified [13, 14]. In our analysis, the final model had a solution with two classes, as it was the model that best fits the data and, thus, more adequate to our population. Also, and according to the current user notes of the scale, two categories can be used (food security (high and marginal food security) vs. food insecurity (low and very low food security)) [14], reflecting the appropriateness of our findings.

The most endorsed items were those related to worrying about food at home run out and meals only including cheap foods because the family did not have enough money, which was in agreement with the results from previous studies [12, 13, 43, 44].

The mean in the food security class was lower than in food insecurity class, representing those individuals were less likely to answer affirmatively. Even that, the thresholds for the items 1 and 3 were higher than in the food insecurity class, which meant that the individuals in the food security class provide affirmative responses to the items 1 and 3 above to the expected.

Our findings on the relationship between socio-demographic characteristics and food insecurity were in accordance with previous studies [3, 33–35, 38, 45], which supports the construct validity of the Self-Administered Food Security Survey Module for Children.

In the children of Generation XXI birth cohort, based on the estimated posterior probabilities, the prevalence of food insecurity was 17.6%. Using the same scale and its recommended thresholds [14], in Western Australian children aged 9 to 13 years old, a prevalence of 20.1% of food insecurity was reported [44]. In another study performed among *colonias* along the Texas-Mexico border, 64.0% of children reported food insecurity [12]. These two studies [12, 44] reported a higher prevalence of food insecurity than ours, which can be justified since they included more disadvantaged populations, while we use a population-based sample, including different socioeconomic strata.

According to the proposed raw score classification of the scale [14], children could be classified as having high food security, marginal food security, low food security, and very low food security, if the raw score is zero, one, two to five and six to nine affirmative responses, respectively, of which, low and very low food security correspond to food insecurity. Based on that classification, the prevalence of food insecurity estimated in our sample would be 28.5%, much higher than the prevalence we estimated (17.6%). However, considering the higher proportion of affirmative response in the item 1 (53%), the prevalence of food insecurity identified using the scale guidelines could be inflated, which justifies the relevance of determining appropriate cut-offs for Portuguese children.

In accordance with our results, for a raw score of two or three affirmative responses, the individuals can be classified differently, as food secure or food insecure, depending on the responses to the items 1 and 3.

The classification tree showed very good accuracy, and so, confirmed that it is possible to construct a simple tool to identify the food security status classes.

To the best of our knowledge, this is the first study in Portugal validating the Self-Administered Food Security Survey Module for Children and thus, stating the prevalence of food insecurity in children based on its own reporting. Evaluating food insecurity experienced by children would enable to assess the determinants and consequences of food insecurity for children, based on its own perceptions, as there is evidence that children were capable to indicate their own food insecurity experience and perceptions [46], and they are generally the best reporters of their own experiences [46].

Furthermore, children answered the Self-Administered Food Security Survey Module for Children separately from the caregivers, reducing the possibility of caregiver’s influence and bias. Also, we used data from a large population-based birth cohort of children with the representation of different socioeconomic strata. This study was also strengthened by the use of classification tree [30], which use has been increasing in the public health area [47], and that enables quick and useful visualization of the identified groups, that shared similar characteristics.

Nevertheless, some limitations should be pointed out. As food insecurity is a sensitive issue, the possibility of social desirability bias and an underestimation of the prevalence of food insecurity cannot be disposed of. However, because of the self-administered nature of the scale and because children answered separately from the parents, its possibility is diminished, as aforementioned.

The Self-Administered Food Security Survey Module for Children was originally designed to be applied to children aged 12 years or more, and in our study, children were, on average, 11 years of age. Nonetheless, previous evidence states that children aged 6 years of age are capable of reporting their food security status [12], thus we do not expect that this had influenced or limited the generalization of our findings. In addition, despite food insecurity demonstrates a range in severity [48] and four food security categories were identified in the original scale [14], in our sample, the best model identified only two food security status categories. Finally, notwithstanding the possibility of recall bias, as the scale items were related to the previous month (30-day reference period, as recommended) [14], the impact of this bias may not be that important.

## Conclusion

Using MLTA, our results sustain that the Self-Administered Food Security Survey Module for Children is a reliable and valid scale for the identification of food insecurity among Portuguese individual children. Classifying individuals into groups and, simultaneously, accounting for the differences within groups, allowed the distinction of two food security status classes for Portuguese children, enhancing the understanding on how the scale works in this specific setting. Moreover, the parameterization of our final model was identical to the sum of the items used in the classification tree.

In line with other recent studies [41, 42], this study supported the appropriateness of using MLTA, having as advantage better performance [16].

The Self-Administered Food Security Survey Module for Children can contribute to the identification of food insecurity, providing evidence-based knowledge for public health policy development based on children’ reports of food insecurity.

## Supporting information

S1 Fig Diagrams of the latent variables modelling tested. The figure describes the models of A) Latent Class Analysis, in which the circumference includes the latent class variable; B) Latent Trait Analysis, in which, the boxes indicate the observed items of the food security survey module and the circumference represents the trait/factor, indicating the correlations between the items; C) Mixture Latent Class Analysis, class invariant item parameters, and D) Mixture Latent Class Analysis, class variant item parameters, in which the arrows from the latent class (c) vary according to the latent class membership.
(TIF)

S1 Appendix Thresholds, unstandardized factor loadings, mean, variance and standardized factor loadings according to the different models tested. (DOCX)

S2 Appendix Response pattern frequencies and chi-square contributions. (DOCX)
