# Supplementary figures and images for: Application of the mixture item response theory model to the Self-Administered Food Security Survey Module for Children
Source: PLoS One. 2020 Jan 23;15(1):e0228099. doi: 10.1371/journal.pone.0228099 (PMC6977726; doi:10.1371/journal.pone.0228099)

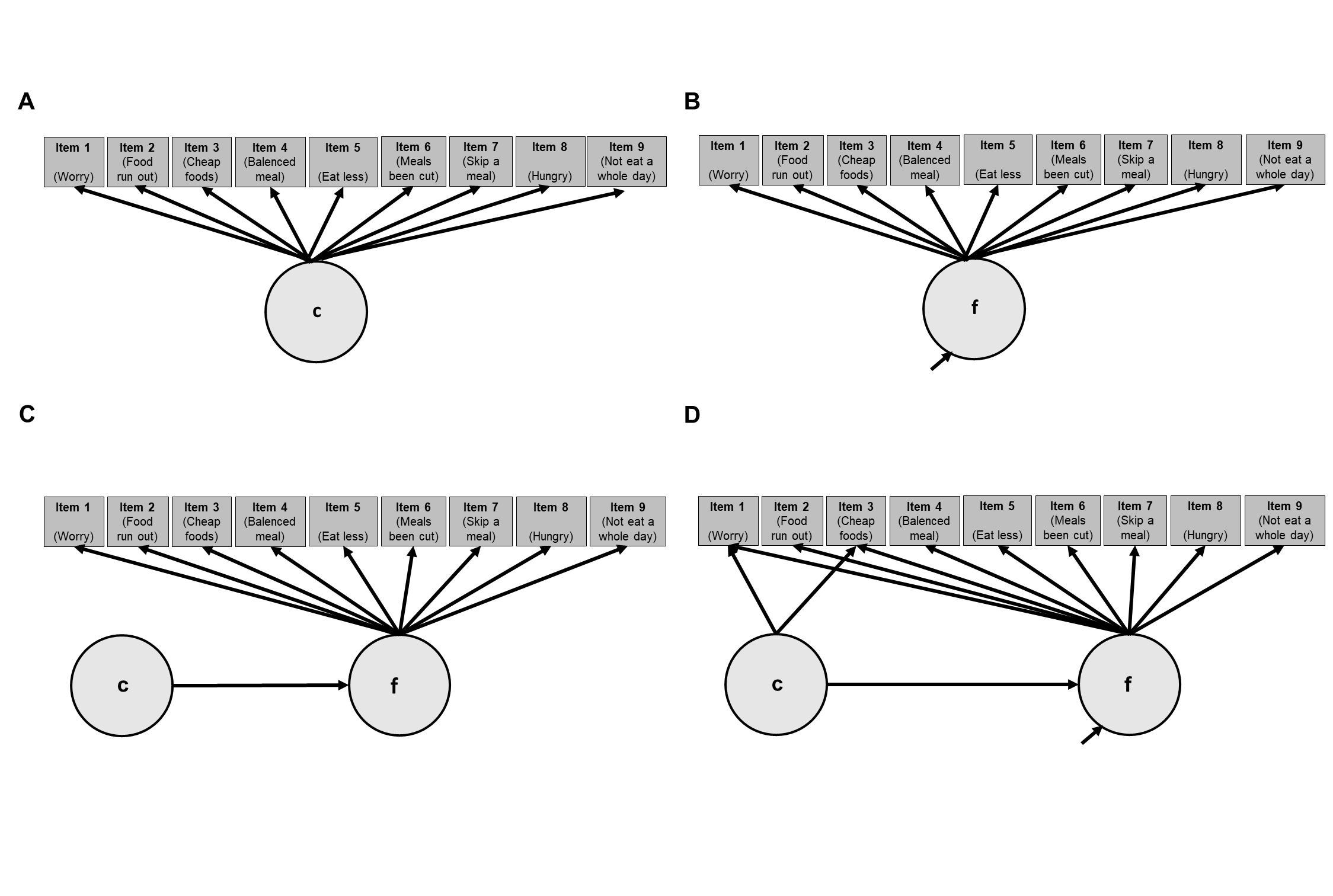

Supplement: S1 Fig — The figure describes the models of A) Latent Class Analysis, in which the circumference includes the latent class variable; B) Latent Trait Analysis, in which, the boxes indicate the observed items of the food security survey module and the circumference represents the trait/factor, indicating the correlations between the items; C) Mixture Latent Class Analysis, class invariant item parameters, and D) Mixture Latent Class Analysis, class variant item parameters, in which the arrows from the latent class (c) vary according to the latent class membership. (TIF) [file pone.0228099.s001.tif]
