# Supplementary material for: Application of the mixture item response theory model to the Self-Administered Food Security Survey Module for Children
Source: PLoS One. 2020 Jan 23;15(1):e0228099. doi: 10.1371/journal.pone.0228099 (PMC6977726; doi:10.1371/journal.pone.0228099)
Supplement: S1 Appendix — (DOCX) [file pone.0228099.s002.docx]

|  | **LCA**  3 classes | | | | | |  | **LTA**  1 f * | | |
| --- | --- | --- | --- | --- | --- | --- | --- | --- | --- | --- |
|  | **Class 1** | | **Class 2** | | **Class 3** | |  |  |  |  |
| % | 64.3 | | 30.2 | | 5.5 | |  |  |  |  |
| **Items** | **%** | **Ƭ**  **(s.e.)** | **%** | **Ƭ**  **(s.e.)** | **%** | **Ƭ**  **(s.e.)** |  | **Ƭ**  **(s.e.)** | ***β***  **(s.e.)** | **β***  **(s.e.)** |
| Item 1 | 38.4 | 0.472  (0.096) | 77.5 | -1.236  (0.202) | 89.2 | -2.111  (0.447) |  | -0.185  (0.066) | 1.711  (0.060) | 0.686  (0.013) |
| Item 2 | 0.7 | 4.889  (0.774) | 15.8 | 1.671  (0.240) | 62.4 | -0.505  (0.419) |  | 3.349  (0.109) | 1.711  (0.060) | 0.686  (0.013) |
| Item 3 | 6.9 | 2.602  (0.314) | 57.3 | -0.293  (0.242) | 78.3 | -1.285  (0.317) |  | 1.551  (0.077) | 1.711  (0.060) | 0.686  (0.013) |
| Item 4 | 0.6 | 5.030  (0.835) | 17.6 | 1.541  (0.272) | 59.6 | -0.387  (0.296) |  | 3.296  (0.107) | 1.711  (0.060) | 0.686  (0.013) |
| Item 5 | 0.0 | 15.000  (-) | 3.7 | 3.258  (0.514) | 55.0 | -0.199  (0.465) |  | 4.332  (0.130) | 1.711  (0.060) | 0.686  (0.013) |
| Item 6 | 0.0 | 15.000  (-) | 11.8 | 2.014  (0.334) | 51.6 | -0.062  (0.290) |  | 3.771  (0.117) | 1.711  (0.060) | 0.686  (0.013) |
| Item 7 | 0.0 | 7.797  (4.060) | 2.7 | 3.570  (0.494) | 26.4 | 1.026  (0.327) |  | 5.038  (0.167) | 1.711  (0.060) | 0.686  (0.013) |
| Item 8 | 1.2 | 4.399  (0.418) | 9.6 | 2.241  (0.195) | 26.9 | 0.999  (0.330) |  | 4.049  (0.140) | 1.711  (0.060) | 0.686  (0.013) |
| Item 9 | 0.0 | 15.000  (-) | 0.3 | 5.836  (1.111) | 6.1 | 2.733  (0.486) |  | 6.897  (0.348) | 1.711  (0.060) | 0.686  (0.013) |
| **Mean (s.e.)** | - | | - | | - | |  | 0 (-) | | |
| **Variance (s.e.)** | - | | - | | - | |  | 1 (-) | | |
| f, Factor; LCA, Latent Class Analysis; LTA, Latent Trait Analysis; s.e., Standard error; Ƭ, Threshold;  *β*, Unstandardized factor loading; β*, Standardized factor loading  * The same model as the Mixture Latent Trait Analysis 1 factor 1 class | | | | | | | | | | |

**S1 Appendix:** Thresholds, unstandardized factor loadings, mean, variance and standardized factor loadings according to the different models tested

|  | **MLTA**  1 f 2 classes (Model 1) | | | | | |  | **MLTA**  1 f 2 classes (Model 3) | | | | | |  | **MLTA**  1 f 2 classes (Model 4) | | | | | |
| --- | --- | --- | --- | --- | --- | --- | --- | --- | --- | --- | --- | --- | --- | --- | --- | --- | --- | --- | --- | --- |
|  | **Class 1** | | | **Class 2** | | |  | **Class 1** | | | **Class 2** | | |  | **Class 1** | | | **Class 2** | | |
| **%** | 41.8 | | | 58.2 | | |  | 74.5 | | | 25.4 | | |  | 83.0 | | | 17.0 | | |
| **Items** | **Ƭ**  **(s.e.)** | ***β***  **(s.e.)** | **β***  **(s.e.)** | **Ƭ**  **(s.e.)** | ***β***  **(s.e.)** | **β***  **(s.e.)** |  | **Ƭ**  **(s.e.)** | ***β***  **(s.e.)** | **β***  **(s.e.)** | **Ƭ**  **(s.e.)** | ***β***  **(s.e.)** | **β***  **(s.e.)** |  | **Ƭ**  **(s.e.)** | ***β***  **(s.e.)** | **β***  **(s.e.)** | **Ƭ**  **(s.e.)** | ***β***  **(s.e.)** | **β***  **(s.e.)** |
| Item 1 | 0.544  (0.252) | 1  (-) | 0.629 (0.042) | 0.544  (0.252) | 1  (-) | 0.483  (-) |  | 0.213 (0.092) | 1  (-) | 0.483 (-) | -1.296 (0.195) | 1  (-) | 0.483  (-) |  | 0.044 (0.119) | 1  (-) | 0.618 (0.018) | -1.118 (0.282) | 1  (-) | 0.483 (-) |
| Item 2 | 4.047  (0.274) | 1  (-) | 0.629 (0.042) | 4.047  (0.274) | 1  (-) | 0.483  (-) |  | 4.269 (0.388) | 1  (-) | 0.483 (-) | 1.158 (0.227) | 1  (-) | 0.483  (-) |  | 4.046 (0.267) | 1  (-) | 0.618 (0.018) | 0.918 (0.384) | 1  (-) | 0.483 (-) |
| Item 3 | 2.226  (0.272) | 1  (-) | 0.629 (0.042) | 2.226  (0.272) | 1  (-) | 0.483  (-) |  | 2.055 (0.174) | 1  (-) | 0.483 (-) | -0.412 (0.202) | 1  (-) | 0.483  (-) |  | 1.998 (0.198) | 1  (-) | 0.618 (0.018) | -0.644 (0.291) | 1  (-) | 0.483 (-) |
| Item 4 | 3.993  (0.274) | 1  (-) | 0.629 (0.042) | 3.993  (0.274) | 1  (-) | 0.483  (-) |  | 4.208 (0.397) | 1  (-) | 0.483 (-) | 1.105 (0.216) | 1  (-) | 0.483  (-) |  | 4.040 (0.300) | 1  (-) | 0.618 (0.018) | 0.819 (0.344) | 1  (-) | 0.483 (-) |
| Item 5 | 5.043  (0.291) | 1  (-) | 0.629 (0.042) | 5.043  (0.291) | 1  (-) | 0.483  (-) |  | 154.668 (-) | 1  (-) | 0.483 (-) | 1.952 (0.274) | 1  (-) | 0.483  (-) |  | 5.919 (0.845) | 1  (-) | 0.618 (0.018) | 1.592 (0.417) | 1  (-) | 0.483 (-) |
| Item 6 | 4.475  (0.279) | 1  (-) | 0.629 (0.042) | 4.475  (0.279) | 1  (-) | 0.483  (-) |  | 6.238 (2.138) | 1  (-) | 0.483 (-) | 1.377 (0.232) | 1  (-) | 0.483  (-) |  | 5.520 (1.523) | 1  (-) | 0.618 (0.018) | 0.881 (0.380) | 1  (-) | 0.483 (-) |
| Item 7 | 5.754  (0.305) | 1  (-) | 0.629 (0.042) | 5.754  (0.305) | 1  (-) | 0.483  (-) |  | 7.228 (2.038) | 1  (-) | 0.483 (-) | 2.746 (0.256) | 1  (-) | 0.483  (-) |  | 6.173 (0.794) | 1  (-) | 0.618 (0.018) | 2.471 (0.323) | 1  (-) | 0.483 (-) |
| Item 8 | 4.757  (0.283) | 1  (-) | 0.629 (0.042) | 4.757  (0.283) | 1  (-) | 0.483  (-) |  | 4.051 (0.243) | 1  (-) | 0.483 (-) | 2.303 (0.194) | 1  (-) | 0.483  (-) |  | 4.145 (0.233) | 1  (-) | 0.618 (0.018) | 2.270 (0.308) | 1  (-) | 0.483 (-) |
| Item 9 | 7.614  (0.453) | 1  (-) | 0.629 (0.042) | 7.614  (0.453) | 1  (-) | 0.483  (-) |  | 5.964 (0.335) | 1  (-) | 0.483 (-) | 5.964 (0.335) | 1  (-) | 0.483  (-) |  | 6.393 (0.330) | 1  (-) | 0.618 (0.018) | 6.393 (0.330) | 1  (-) | 0.483 (-) |
| **Mean (s.e.)** | 1.893 (1.169) | | | 0 (-) | | |  | 0 (-) | | | 0 (-) | | |  | 0 (-) | | | 0 (-) | | |
| **Variance (s.e.)** | 2.151 (1.389) | | | 1 (-) | | |  | 1 (-) | | | 1 (-) | | |  | 2.032 (0.535) | | | 1 (-) | | |
| f, Factor; MLTA. Mixture Latent Trait Analysis; s.e., Standard error; Ƭ. Threshold; *β*, Unstandardized factor loading; β*, Standardized factor loading | | | | | | | | | | | | | | | | | | | | |

|  | **MLTA**  1 f 2 classes (Model 5) | | | | | |  | **MLTA**  1 f 2 classes (Model 6) | | | | | |
| --- | --- | --- | --- | --- | --- | --- | --- | --- | --- | --- | --- | --- | --- |
|  | **Class 1** | | | **Class 2** | | |  | **Class 1** | | | **Class 2** | | |
| **%** |  | 82.0 |  |  | 18.0 |  |  | 92.2 | | | 7.8 | | |
| **Items** | **Ƭ**  **(s.e.)** | ***β***  **(s.e.)** | **β***  **(s.e.)** | **Ƭ**  **(s.e.)** | ***β***  **(s.e.)** | **β***  **(s.e.)** |  | **Ƭ**  **(s.e.)** | ***β***  **(s.e.)** | **β***  **(s.e.)** | **Ƭ**  **(s.e.)** | ***β***  **(s.e.)** | **β***  **(s.e.)** |
| Item 1 | 0.111 (0.092) | 1  (-) | 0.566 (0.013) | -1.346 (0.219) | 1  (-) | 0.426 (0.034) |  | -0.334 (0.265) | 2.113 (1.178) | 0.759 (0.179) | 1.036 (2.479) | 1.187 (1.465) | 0.548 (0.473) |
| Item 2 | 4.100 (0.264) | 1  (-) | 0.566 (0.013) | 0.775 (0.338) | 1  (-) | 0.426 (0.034) |  | 3.784 (0.279) | 1.850 (0.290) | 0.714 (0.055) | 1.878 (1.643) | 2.732 (2.262) | 0.833 (0.211) |
| Item 3 | 1.976 (0.166) | 1  (-) | 0.566 (0.013) | -0.716 (0.216) | 1  (-) | 0.426 (0.034) |  | 1.986 (1.020) | 1.971 (1.008) | 0.736 (0.173) | -0.814 (0.486) | -0.540 (0.765) | -0.285 (0.371) |
| Item 4 | 4.064 (0.293) | 1  (-) | 0.566 (0.013) | 0.707 (0.310) | 1  (-) | 0.426 (0.034) |  | 3.886 (0.400) | 1.990 (0.260) | 0.739 (0.044) | 1.073 (1.105) | 1.076 (1.076) | 0.510 (0.377) |
| Item 5 | 8.131 (4.202) | 1  (-) | 0.566 (0.013) | 1.403 (0.383) | 1  (-) | 0.426 (0.034) |  | 6.170 (0.665) | 2.827 (0.512) | 0.842 (0.044) | 4.382 (3.654) | 3.625 (2.619) | 0.894 (0.129) |
| Item 6 | 5.140 (0.774) | 1  (-) | 0.566 (0.013) | 0.974 (0.262) | 1  (-) | 0.426 (0.034) |  | 5.539 (1.093) | 2.910 (0.646) | 0.849 (0.053) | 1.433 (0.555) | 0.072 (1.623) | 0.040 (0.892) |
| Item 7 | 6.483 (0.822) | 1  (-) | 0.566 (0.013) | 2.323 (0.342) | 1  (-) | 0.426 (0.034) |  | 6.002 (0.627) | 2.305 (0.425) | 0.786 (0.055) | 3.976 (1.776) | 1.616 (1.424) | 0.665 (0.327) |
| Item 8 | 4.146 (0.225) | 1  (-) | 0.566 (0.013) | 1.987 (0.309) | 1  (-) | 0.426 (0.034) |  | 3.791 (0.350) | 1.176 (0.205) | 0.544 (0.067) | 1.569 (0.962) | 0.750 (0.461) | 0.382 (0.201) |
| Item 9 | 256.389  (-) | 1  (-) | 0.566 (0.013) | 4.070 (0.472) | 1  (-) | 0.426 (0.034) |  | 7.676 (0.957) | 1.993 (0.507) | 0.740 (0.085) | 7.676 (0.957) | 3.224 (1.873) | 0.872 (0.122) |
| **Mean (s.e.)** | 0 (-) | | | 0 (-) | | |  | 0 (-) | | | 0 (-) | | |
| **Variance (s.e.)** | 1.554 (0.253) | | | 0.728 (0.242) | | |  | 1 (-) | | | 1 (-) | | |
| f, Factor; MLTA. Mixture Latent Trait Analysis; s.e., Standard error; Ƭ. Threshold; *β*, Unstandardized factor loading; β*, Standardized factor loading | | | | | | | | | | | | | |

|  | **MLTA**  1 f 3 classes | | | | | | | | | |
| --- | --- | --- | --- | --- | --- | --- | --- | --- | --- | --- |
|  | **Class 1** | | | **Class 2** | | | **Class 3** | | | |
| % | 14.7 | | | 81.9 | | | 3.4 | | | |
| **Items** | **Ƭ**  **(s.e.)** | ***β***  **(s.e.)** | **β***  **(s.e.)** | **Ƭ**  **(s.e.)** | ***β***  **(s.e.)** | **β***  **(s.e.)** | **Ƭ**  **(s.e.)** | ***β***  **(s.e.)** | **β***  **(s.e.)** |  |
| Item 1 | -0.637 (2.961) | 1  (-) | 0.483  (-) | -2.856  (1.376) | 1  (-) | 0.672  (0.120) | 0.574 (2.310) | 1  (-) | 0.468  (0.052) |  |
| Item 2 | 1.407 (2.033) | 1  (-) | 0.483  (-) | 1.407  (2.033) | 1  (-) | 0.672  (0.120) | 1.407 (2.033) | 1  (-) | 0.468  (0.052) |  |
| Item 3 | -1.361 (2.458) | 1  (-) | 0.483  (-) | -0.505  (2.729) | 1  (-) | 0.672  (0.120) | 2.088 (1.201) | 1  (-) | 0.468  (0.052) |  |
| Item 4 | 1.351 (2.033) | 1  (-) | 0.483  (-) | 1.351  (2.033) | 1  (-) | 0.672  (0.120) | 1.351 (2.033) | 1  (-) | 0.468  (0.052) |  |
| Item 5 | 2.441 (2.036) | 1  (-) | 0.483  (-) | 2.441  (2.036) | 1  (-) | 0.672  (0.120) | 2.441 (2.036) | 1  (-) | 0.468  (0.052) |  |
| Item 6 | 1.855 (2.022) | 1  (-) | 0.483  (-) | 1.855  (2.022) | 1  (-) | 0.672  (0.120) | 1.855 (2.022) | 1  (-) | 0.468  (0.052) |  |
| Item 7 | 3.167 (2.043) | 1  (-) | 0.483  (-) | 3.167  (2.043) | 1  (-) | 0.672  (0.120) | 3.167 (2.043) | 1  (-) | 0.468  (0.052) |  |
| Item 8 | 2.147 (2.021) | 1  (-) | 0.483  (-) | 2.147  (2.021) | 1  (-) | 0.672  (0.120) | 2.147 (2.021) | 1  (-) | 0.468  (0.052) |  |
| Item 9 | 5.041 (2.113) | 1  (-) | 0.483  (-) | 5.041  (2.113) | 1  (-) | 0.672  (0.120) | 5.041 (2.113) | 1  (-) | 0.468  (0.052) |  |
| **Mean (s.e.)** | 0 (-) | | | -2.824 (2.108) | | | 1.562 (1.266) | | | |
| **Variance (s.e.)** | 1 (-) | | | 2.715 (5.830) | | | 0.922 (0.506) | | | |
| f, Factor; MLTA. Mixture Latent Trait Analysis; s.e., Standard error; Ƭ. Threshold; *β*. Unstandardized factor loading; β*, Standardized factor loading | | | | | | | | | | |
