# Supplementary material for: Application of the mixture item response theory model to the Self-Administered Food Security Survey Module for Children
Source: PLoS One. 2020 Jan 23;15(1):e0228099. doi: 10.1371/journal.pone.0228099 (PMC6977726; doi:10.1371/journal.pone.0228099)
Supplement: S2 Appendix — (DOCX) [file pone.0228099.s003.docx]

| **S2 Appendix:** Response pattern frequencies and chi-square contributions | | | | | | |
| --- | --- | --- | --- | --- | --- | --- |
| **Response Pattern** | **Frequency** | | **Standardized Residual (z-score)** | **Chi-square Pearson** | **Contribution Log-likelihood** | **Deleted** |
|  | **Observed** | **Estimated** |  |  |  |  |
| 1 | 799 | 799.99 | -0.04 | 0 | -1.99 |  |
| 2 | 1 | 0.86 | 0.15 | 0.02 | 0.29 |  |
| 3 | 11 | 12.91 | -0.53 | 0.28 | -3.52 |  |
| 4 | 30 | 26.46 | 0.69 | 0.47 | 7.53 |  |
| 5 | 98 | 93.09 | 0.52 | 0.26 | 10.07 |  |
| 6 | 9 | 7.88 | 0.4 | 0.16 | 2.4 |  |
| 7 | 1 | 1.44 | -0.37 | 0.14 | -0.73 |  |
| 8 | 586 | 581.69 | 0.21 | 0.03 | 8.65 |  |
| 9 | 191 | 201.06 | -0.75 | 0.5 | -19.62 |  |
| 10 | 12 | 16.3 | -1.07 | 1.13 | -7.35 |  |
| 11 | 2 | 0.05 | 8.92 | 79.55 | 14.93 |  |
| 12 | 27 | 26.97 | 0 | 0 | 0.05 |  |
| 13 | 20 | 16.92 | 0.75 | 0.56 | 6.69 |  |
| 14 | 31 | 28 | 0.57 | 0.32 | 6.3 |  |
| 15 | 2 | 2.32 | -0.21 | 0.04 | -0.59 |  |
| 16 | 1 | 0.14 | 2.27 | 5.14 | 3.89 |  |
| 17 | 3 | 2.45 | 0.35 | 0.12 | 1.21 |  |
| 18 | 1 | 0.41 | 0.93 | 0.87 | 1.81 |  |
| 19 | 4 | 5.34 | -0.58 | 0.34 | -2.32 |  |
| 20 | 15 | 12.66 | 0.66 | 0.43 | 5.09 |  |
| 21 | 13 | 6.88 | 2.34 | 5.44 | 16.55 |  |
| 22 | 4 | 8.26 | -1.48 | 2.19 | -5.8 |  |
| 23 | 7 | 4.99 | 0.9 | 0.81 | 4.74 |  |
| 24 | 1 | 0.81 | 0.21 | 0.04 | 0.41 |  |
| 25 | 12 | 13.66 | -0.45 | 0.2 | -3.11 |  |
| 26 | 13 | 6.18 | 2.75 | 7.54 | 19.35 |  |
| 27 | 5 | 4.46 | 0.25 | 0.06 | 1.14 |  |
| 28 | 29 | 25.49 | 0.7 | 0.48 | 7.49 |  |
| 29 | 11 | 4.76 | 2.86 | 8.18 | 18.43 |  |
| 30 | 3 | 3.64 | -0.33 | 0.11 | -1.15 |  |
| 31 | 2 | 1.74 | 0.2 | 0.04 | 0.56 |  |
| 32 | 3 | 1.14 | 1.75 | 3.05 | 5.82 |  |
| 33 | 1 | 0.15 | 2.21 | 4.89 | 3.82 |  |
| 34 | 4 | 7.44 | -1.26 | 1.59 | -4.97 |  |
| 35 | 2 | 1.54 | 0.37 | 0.14 | 1.05 |  |
| 36 | 8 | 10.76 | -0.84 | 0.71 | -4.74 |  |
| 37 | 3 | 5.15 | -0.95 | 0.9 | -3.24 |  |
| 38 | 1 | 0.14 | 2.27 | 5.13 | 3.89 |  |
| 39 | 1 | 0.64 | 0.46 | 0.21 | 0.9 |  |
| 40 | 6 | 9.46 | -1.13 | 1.27 | -5.47 |  |
| 41 | 6 | 3.56 | 1.29 | 1.67 | 6.26 |  |
| 42 | 2 | 1.74 | 0.2 | 0.04 | 0.56 |  |
| 43 | 2 | 0.9 | 1.17 | 1.36 | 3.21 |  |
| 44 | 8 | 2.99 | 2.9 | 8.4 | 15.75 |  |
| 45 | 1 | 0.32 | 1.2 | 1.44 | 2.27 |  |
| 46 | 1 | 0.49 | 0.72 | 0.52 | 1.41 |  |
| 47 | 2 | 1.91 | 0.06 | 0 | 0.18 |  |
| 48 | 2 | 4.86 | -1.3 | 1.69 | -3.55 |  |
| 49 | 7 | 9.11 | -0.7 | 0.49 | -3.7 |  |
| 50 | 1 | 0.86 | 0.15 | 0.02 | 0.29 |  |
| 51 | 1 | 3.11 | -1.2 | 1.43 | -2.27 |  |
| 52 | 7 | 6.5 | 0.2 | 0.04 | 1.04 |  |
| 53 | 3 | 2.26 | 0.5 | 0.25 | 1.71 |  |
| 54 | 3 | 3.01 | -0.01 | 0 | -0.03 |  |
| 55 | 2 | 1.78 | 0.17 | 0.03 | 0.47 |  |
| 56 | 1 | 0.02 | 6.35 | 40.28 | 7.49 |  |
| 57 | 2 | 0.11 | 5.66 | 32.09 | 11.56 |  |
| 58 | 1 | 0.66 | 0.42 | 0.17 | 0.83 |  |
| 59 | 2 | 0.3 | 3.14 | 9.85 | 7.65 |  |
| 60 | 4 | 4.63 | -0.29 | 0.09 | -1.17 |  |
| 61 | 17 | 12.2 | 1.38 | 1.89 | 11.29 |  |
| 62 | 2 | 0.3 | 3.11 | 9.68 | 7.6 |  |
| 63 | 1 | 0.59 | 0.54 | 0.29 | 1.07 |  |
| 64 | 5 | 3.85 | 0.59 | 0.35 | 2.62 |  |
| 65 | 3 | 2.39 | 0.4 | 0.16 | 1.37 |  |
| 66 | 1 | 0.09 | 3.11 | 9.66 | 4.9 |  |
| 67 | 4 | 1.81 | 1.63 | 2.67 | 6.36 |  |
| 68 | 1 | 2.42 | -0.91 | 0.83 | -1.76 |  |
| 69 | 1 | 0.67 | 0.4 | 0.16 | 0.8 |  |
| 70 | 1 | 1.55 | -0.44 | 0.19 | -0.87 |  |
| 71 | 2 | 1.15 | 0.79 | 0.63 | 2.21 |  |
| 72 | 1 | 1.16 | -0.15 | 0.02 | -0.29 |  |
| 73 | 1 | 0.21 | 1.75 | 3.06 | 3.16 |  |
| 74 | 1 | 0.52 | 0.66 | 0.44 | 1.3 |  |
| 75 | 2 | 1.22 | 0.71 | 0.5 | 1.98 |  |
| 76 | 1 | 0.11 | 2.68 | 7.16 | 4.4 |  |
| 77 | 1 | 0.36 | 1.08 | 1.17 | 2.07 |  |
| 78 | 1 | 0.5 | 0.7 | 0.5 | 1.38 |  |
| 79 | 2 | 1.09 | 0.87 | 0.76 | 2.43 |  |
| 80 | 1 | 0.4 | 0.95 | 0.91 | 1.84 |  |
| 81 | 1 | 0.79 | 0.24 | 0.06 | 0.48 |  |
| 82 | 1 | 0.03 | 5.31 | 28.18 | 6.81 |  |
| 83 | 1 | 0.19 | 1.85 | 3.44 | 3.32 |  |
| 84 | 1 | 1.05 | -0.05 | 0 | -0.1 |  |
| 85 | 3 | 1.88 | 0.81 | 0.66 | 2.79 |  |
| 86 | 1 | 4 | -1.5 | 2.25 | -2.77 |  |
| 87 | 3 | 1.11 | 1.8 | 3.23 | 5.98 |  |
| 88 | 1 | 0.39 | 0.96 | 0.93 | 1.86 |  |
| 89 | 4 | 2.32 | 1.1 | 1.21 | 4.34 |  |
| 90 | 1 | 0.55 | 0.6 | 0.36 | 1.18 |  |
| 91 | 1 | 1.08 | -0.08 | 0.01 | -0.15 |  |
| 92 | 1 | 1.17 | -0.16 | 0.03 | -0.32 |  |
| 93 | 1 | 0.41 | 0.91 | 0.83 | 1.77 |  |
| 94 | 2 | 1.2 | 0.73 | 0.53 | 2.04 |  |
| 95 | 1 | 0.07 | 3.5 | 12.26 | 5.3 |  |
| 96 | 1 | 0 | 14.63 | 213.89 | 10.75 | DELETED |
| 97 | 1 | 1.83 | -0.62 | 0.38 | -1.21 |  |
| 98 | 1 | 0.7 | 0.36 | 0.13 | 0.73 |  |
| 99 | 2 | 2.41 | -0.26 | 0.07 | -0.74 |  |
| 100 | 1 | 0.14 | 2.3 | 5.29 | 3.94 |  |
| 101 | 2 | 0.74 | 1.47 | 2.15 | 3.98 |  |
| 102 | 1 | 0.81 | 0.22 | 0.05 | 0.43 |  |
| 103 | 1 | 0.54 | 0.62 | 0.39 | 1.22 |  |
| 104 | 1 | 0.64 | 0.46 | 0.21 | 0.91 |  |
| 105 | 1 | 0.1 | 2.83 | 8 | 4.59 |  |
| 106 | 1 | 0.28 | 1.36 | 1.84 | 2.54 |  |
| 107 | 1 | 0.57 | 0.56 | 0.32 | 1.11 |  |
| 108 | 1 | 0.3 | 1.29 | 1.66 | 2.42 |  |
| 109 | 1 | 2.26 | -0.84 | 0.7 | -1.63 |  |
| 110 | 1 | 0.9 | 0.11 | 0.01 | 0.22 |  |
| 111 | 1 | 1.69 | -0.53 | 0.28 | -1.04 |  |
| 112 | 1 | 0.86 | 0.15 | 0.02 | 0.3 |  |
| 113 | 1 | 0.94 | 0.07 | 0 | 0.13 |  |
| 114 | 2 | 0.85 | 1.25 | 1.56 | 3.43 |  |
| 115 | 1 | 3.19 | -1.23 | 1.5 | -2.32 |  |
| 116 | 1 | 0.23 | 1.59 | 2.53 | 2.92 |  |
| 117 | 1 | 0.26 | 1.47 | 2.16 | 2.73 |  |
| 118 | 1 | 0.83 | 0.19 | 0.03 | 0.37 |  |
| 119 | 1 | 0.5 | 0.7 | 0.49 | 1.38 |  |
| 120 | 1 | 4.62 | -1.69 | 2.83 | -3.06 |  |
| 121 | 1 | 0.41 | 0.93 | 0.87 | 1.8 |  |
|  |  |  |  |  |  |  |
| The total Pearson Chi-square contribution from empty cells is 53.23 | | | | | | |

| Univariate Model Fit Information |  |  |  |
| --- | --- | --- | --- |
|  | **Estimated** **Probabilities** | | |
| **Variable** | **H1** | **H0** | **Standardized Residual (z-score)** |
| **ITEM 1** |  |  |  |
| Category 1 | 0.47 | 0.47 | 0.002 |
| Category 2 | 0.53 | 0.53 | -0.002 |
| Univariate Pearson Chi-Square |  |  | 0.000 |
| Univariate Log-Likelihood Chi-Square |  |  | 0.000 |
| **ITEM 2** |  |  |  |
| Category 1 | 0.913 | 0.913 | 0.004 |
| Category 2 | 0.087 | 0.087 | -0.004 |
| Univariate Pearson Chi-Square |  |  | 0.000 |
| Univariate Log-Likelihood Chi-Square |  |  | 0.000 |
| **ITEM 3** |  |  |  |
| Category 1 | 0.74 | 0.74 | 0.000 |
| Category 2 | 0.26 | 0.26 | 0.000 |
| Univariate Pearson Chi-Square |  |  | 0.000 |
| Univariate Log-Likelihood Chi-Square |  |  | 0.000 |
| **ITEM 4** |  |  |  |
| Category 1 | 0.91 | 0.91 | 0.005 |
| Category 2 | 0.09 | 0.09 | -0.005 |
| Univariate Pearson Chi-Square |  |  | 0.000 |
| Univariate Log-Likelihood Chi-Square |  |  | 0.000 |
| **ITEM 5** |  |  |  |
| Category 1 | 0.959 | 0.959 | 0.004 |
| Category 2 | 0.041 | 0.041 | -0.004 |
| Univariate Pearson Chi-Square |  |  | 0.000 |
| Univariate Log-Likelihood Chi-Square |  |  | 0.000 |
| **ITEM 6** |  |  |  |
| Category 1 | 0.936 | 0.936 | 0.007 |
| Category 2 | 0.064 | 0.064 | -0.007 |
| Univariate Pearson Chi-Square |  |  | 0.000 |
| Univariate Log-Likelihood Chi-Square |  |  | 0.000 |
| **ITEM 7** |  |  |  |
| Category 1 | 0.977 | 0.977 | 0.000 |
| Category 2 | 0.023 | 0.023 | 0.000 |
| Univariate Pearson Chi-Square |  |  | 0.000 |
| Univariate Log-Likelihood Chi-Square |  |  | 0.000 |
| **ITEM 8** |  |  |  |
| Category 1 | 0.948 | 0.948 | 0.005 |
| Category 2 | 0.052 | 0.052 | -0.005 |
| Univariate Pearson Chi-Square |  |  | 0.000 |
| Univariate Log-Likelihood Chi-Square |  |  | 0.000 |
| **ITEM 9** |  |  |  |
| Category 1 | 0.996 | 0.996 | -0.004 |
| Category 2 | 0.004 | 0.004 | 0.004 |
| Univariate Pearson Chi-Square |  |  | 0.000 |
| Univariate Log-Likelihood Chi-Square |  |  | 0.000 |
|  |  |  |  |
| **Overall Univariate Pearson Chi-Square** |  |  | 0.000 |
| **Overall Univariate Log-Likelihood Chi-Square** |  |  | 0.000 |

| Bivariate Model Fit Information | | | | |
| --- | --- | --- | --- | --- |
| **Variable** | **Variable** | **Estimated Probabilities** | | |
|  |  | **H1** | **H0** | **Standardized Residual (z-score)** |
| Item 1 | Item 2 |  |  |  |
| Category 1 | Category 1 | 0.453 | 0.452 | 0.141 |
| Category 1 | Category 2 | 0.017 | 0.018 | -0.514 |
| Category 2 | Category 1 | 0.460 | 0.462 | -0.138 |
| Category 2 | Category 2 | 0.070 | 0.068 | 0.269 |
| Bivariate Pearson Chi-Square | |  |  | 0.348 |
| Bivariate Log-Likelihood Chi-square | |  |  | 0.355 |
| Item 1 | Item 3 |  |  |  |
| Category 1 | Category 1 | 0.401 | 0.404 | -0.284 |
| Category 1 | Category 2 | 0.069 | 0.066 | 0.563 |
| Category 2 | Category 1 | 0.339 | 0.336 | 0.294 |
| Category 2 | Category 2 | 0.191 | 0.194 | -0.354 |
| Bivariate Pearson Chi-Square | |  |  | 0.503 |
| Bivariate Log-Likelihood Chi-square | |  |  | 0.499 |
| Item 1 | Item 4 |  |  |  |
| Category 1 | Category 1 | 0.451 | 0.451 | -0.002 |
| Category 1 | Category 2 | 0.019 | 0.019 | 0.016 |
| Category 2 | Category 1 | 0.459 | 0.459 | 0.005 |
| Category 2 | Category 2 | 0.071 | 0.071 | -0.014 |
| Bivariate Pearson Chi-Square | |  |  | 0.000 |
| Bivariate Log-Likelihood Chi-square | |  |  | 0.000 |
| Item 1 | Item 5 |  |  |  |
| Category 1 | Category 1 | 0.465 | 0.462 | 0.307 |
| Category 1 | Category 2 | 0.005 | 0.008 | -1.707 |
| Category 2 | Category 1 | 0.493 | 0.497 | -0.304 |
| Category 2 | Category 2 | 0.037 | 0.033 | 0.841 |
| Bivariate Pearson Chi-Square | |  |  | 3.671 |
| Bivariate Log-Likelihood Chi-square | |  |  | 4.157 |
| Item 1 | Item 6 |  |  |  |
| Category 1 | Category 1 | 0.458 | 0.457 | 0.073 |
| Category 1 | Category 2 | 0.012 | 0.013 | -0.312 |
| Category 2 | Category 1 | 0.478 | 0.479 | -0.069 |
| Category 2 | Category 2 | 0.052 | 0.051 | 0.152 |
| Bivariate Pearson Chi-Square | |  |  | 0.124 |
| Bivariate Log-Likelihood Chi-square | |  |  | 0.126 |
| Item 1 | Item 7 |  |  |  |
| Category 1 | Category 1 | 0.468 | 0.466 | 0.176 |
| Category 1 | Category 2 | 0.002 | 0.004 | -1.338 |
| Category 2 | Category 1 | 0.509 | 0.511 | -0.176 |
| Category 2 | Category 2 | 0.021 | 0.019 | 0.639 |
| Bivariate Pearson Chi-Square | |  |  | 2.216 |
| Bivariate Log-Likelihood Chi-square | |  |  | 2.549 |
| Item 1 | Item 8 |  |  |  |
| Category 1 | Category 1 | 0.455 | 0.460 | -0.485 |
| Category 1 | Category 2 | 0.015 | 0.010 | 2.417 |
| Category 2 | Category 1 | 0.494 | 0.489 | 0.486 |
| Category 2 | Category 2 | 0.036 | 0.041 | -1.225 |
| Bivariate Pearson Chi-Square | |  |  | 7.467 |
| Bivariate Log-Likelihood Chi-square | |  |  | 6.736 |
| Item 1 | Item 9 |  |  |  |
| Category 1 | Category 1 | 0.470 | 0.469 | 0.026 |
| Category 1 | Category 2 | 0.000 | 0.001 | -0.437 |
| Category 2 | Category 1 | 0.526 | 0.527 | -0.026 |
| Category 2 | Category 2 | 0.004 | 0.003 | 0.203 |
| Bivariate Pearson Chi-Square | |  |  | 0.232 |
| Bivariate Log-Likelihood Chi-square | |  |  | 0.259 |
| Item 2 | Item 3 |  |  |  |
| Category 1 | Category 1 | 0.704 | 0.708 | -0.384 |
| Category 1 | Category 2 | 0.209 | 0.205 | 0.435 |
| Category 2 | Category 1 | 0.036 | 0.032 | 0.994 |
| Category 2 | Category 2 | 0.051 | 0.055 | -0.771 |
| Bivariate Pearson Chi-Square | |  |  | 1.713 |
| Bivariate Log-Likelihood Chi-square | |  |  | 1.690 |
| Item 2 | Item 4 |  |  |  |
| Category 1 | Category 1 | 0.853 | 0.854 | -0.102 |
| Category 1 | Category 2 | 0.061 | 0.060 | 0.157 |
| Category 2 | Category 1 | 0.057 | 0.056 | 0.162 |
| Category 2 | Category 2 | 0.030 | 0.030 | -0.224 |
| Bivariate Pearson Chi-Square | |  |  | 0.098 |
| Bivariate Log-Likelihood Chi-square | |  |  | 0.098 |
| Item 2 | Item 5 |  |  |  |
| Category 1 | Category 1 | 0.894 | 0.888 | 0.896 |
| Category 1 | Category 2 | 0.019 | 0.025 | -1.792 |
| Category 2 | Category 1 | 0.065 | 0.071 | -1.099 |
| Category 2 | Category 2 | 0.022 | 0.016 | 2.24 |
| Bivariate Pearson Chi-Square | |  |  | 9.279 |
| Bivariate Log-Likelihood Chi-square | |  |  | 9.070 |
| Item 2 | Item 6 |  |  |  |
| Category 1 | Category 1 | 0.872 | 0.872 | -0.072 |
| Category 1 | Category 2 | 0.041 | 0.041 | 0.127 |
| Category 2 | Category 1 | 0.064 | 0.064 | 0.106 |
| Category 2 | Category 2 | 0.023 | 0.023 | -0.179 |
| Bivariate Pearson Chi-Square | |  |  | 0.058 |
| Bivariate Log-Likelihood Chi-square | |  |  | 0.058 |
| Item 2 | Item 7 |  |  |  |
| Category 1 | Category 1 | 0.901 | 0.900 | 0.211 |
| Category 1 | Category 2 | 0.012 | 0.014 | -0.539 |
| Category 2 | Category 1 | 0.076 | 0.077 | -0.237 |
| Category 2 | Category 2 | 0.011 | 0.009 | 0.644 |
| Bivariate Pearson Chi-Square | |  |  | 0.753 |
| Bivariate Log-Likelihood Chi-square | |  |  | 0.745 |
| Item 2 | Item 8 |  |  |  |
| Category 1 | Category 1 | 0.876 | 0.881 | -0.678 |
| Category 1 | Category 2 | 0.037 | 0.032 | 1.249 |
| Category 2 | Category 1 | 0.072 | 0.067 | 0.880 |
| Category 2 | Category 2 | 0.015 | 0.019 | -1.610 |
| Bivariate Pearson Chi-Square | |  |  | 4.829 |
| Bivariate Log-Likelihood Chi-square | |  |  | 4.984 |
| Item 2 | Item 9 |  |  |  |
| Category 1 | Category 1 | 0.911 | 0.911 | 0.080 |
| Category 1 | Category 2 | 0.002 | 0.002 | -0.450 |
| Category 2 | Category 1 | 0.084 | 0.085 | -0.083 |
| Category 2 | Category 2 | 0.002 | 0.002 | 0.511 |
| Bivariate Pearson Chi-Square | |  |  | 0.469 |
| Bivariate Log-Likelihood Chi-square | |  |  | 0.465 |
| Item 3 | Item 4 |  |  |  |
| Category 1 | Category 1 | 0.706 | 0.706 | 0.000 |
| Category 1 | Category 2 | 0.033 | 0.033 | -0.001 |
| Category 2 | Category 1 | 0.204 | 0.204 | 0.003 |
| Category 2 | Category 2 | 0.057 | 0.057 | -0.005 |
| Bivariate Pearson Chi-Square | |  |  | 0.000 |
| Bivariate Log-Likelihood Chi-square | |  |  | 0.000 |
| Item 3 | Item 5 |  |  |  |
| Category 1 | Category 1 | 0.727 | 0.726 | 0.092 |
| Category 1 | Category 2 | 0.013 | 0.014 | -0.354 |
| Category 2 | Category 1 | 0.232 | 0.233 | -0.095 |
| Category 2 | Category 2 | 0.029 | 0.028 | 0.244 |
| Bivariate Pearson Chi-Square | |  |  | 0.191 |
| Bivariate Log-Likelihood Chi-square | |  |  | 0.193 |
| Item 3 | Item 6 |  |  |  |
| Category 1 | Category 1 | 0.723 | 0.717 | 0.548 |
| Category 1 | Category 2 | 0.017 | 0.022 | -1.675 |
| Category 2 | Category 1 | 0.213 | 0.219 | -0.593 |
| Category 2 | Category 2 | 0.047 | 0.042 | 1.228 |
| Bivariate Pearson Chi-Square | |  |  | 4.548 |
| Bivariate Log-Likelihood Chi-square | |  |  | 4.743 |
| Item 3 | Item 7 |  |  |  |
| Category 1 | Category 1 | 0.732 | 0.733 | -0.041 |
| Category 1 | Category 2 | 0.008 | 0.007 | 0.213 |
| Category 2 | Category 1 | 0.245 | 0.244 | 0.042 |
| Category 2 | Category 2 | 0.015 | 0.016 | -0.143 |
| Bivariate Pearson Chi-Square | |  |  | 0.067 |
| Bivariate Log-Likelihood Chi-square | |  |  | 0.066 |
| Item 3 | Item 8 |  |  |  |
| Category 1 | Category 1 | 0.715 | 0.722 | -0.718 |
| Category 1 | Category 2 | 0.024 | 0.017 | 2.456 |
| Category 2 | Category 1 | 0.233 | 0.226 | 0.771 |
| Category 2 | Category 2 | 0.027 | 0.034 | -1.774 |
| Bivariate Pearson Chi-Square | |  |  | 9.571 |
| Bivariate Log-Likelihood Chi-square | |  |  | 9.136 |
| Item 3 | Item 9 |  |  |  |
| Category 1 | Category 1 | 0.739 | 0.738 | 0.077 |
| Category 1 | Category 2 | 0.000 | 0.001 | -0.983 |
| Category 2 | Category 1 | 0.257 | 0.257 | -0.078 |
| Category 2 | Category 2 | 0.004 | 0.003 | 0.629 |
| Bivariate Pearson Chi-Square | |  |  | 1.365 |
| Bivariate Log-Likelihood Chi-square | |  |  | 1.632 |
| Item 4 | Item 5 |  |  |  |
| Category 1 | Category 1 | 0.888 | 0.885 | 0.485 |
| Category 1 | Category 2 | 0.022 | 0.025 | -0.985 |
| Category 2 | Category 1 | 0.070 | 0.074 | -0.59 |
| Category 2 | Category 2 | 0.020 | 0.016 | 1.202 |
| Bivariate Pearson Chi-Square | |  |  | 2.717 |
| Bivariate Log-Likelihood Chi-square | |  |  | 2.68 |
| Item 4 | Item 6 |  |  |  |
| Category 1 | Category 1 | 0.871 | 0.870 | 0.098 |
| Category 1 | Category 2 | 0.039 | 0.04 | -0.161 |
| Category 2 | Category 1 | 0.066 | 0.066 | -0.125 |
| Category 2 | Category 2 | 0.024 | 0.024 | 0.195 |
| Bivariate Pearson Chi-Square | |  |  | 0.078 |
| Bivariate Log-Likelihood Chi-square | |  |  | 0.078 |
| Item 4 | Item 7 |  |  |  |
| Category 1 | Category 1 | 0.897 | 0.897 | 0.03 |
| Category 1 | Category 2 | 0.013 | 0.013 | -0.068 |
| Category 2 | Category 1 | 0.080 | 0.080 | -0.033 |
| Category 2 | Category 2 | 0.010 | 0.010 | 0.079 |
| Bivariate Pearson Chi-Square | |  |  | 0.012 |
| Bivariate Log-Likelihood Chi-square | |  |  | 0.012 |
| Item 4 | Item 8 |  |  |  |
| Category 1 | Category 1 | 0.873 | 0.878 | -0.679 |
| Category 1 | Category 2 | 0.037 | 0.032 | 1.276 |
| Category 2 | Category 1 | 0.075 | 0.070 | 0.874 |
| Category 2 | Category 2 | 0.015 | 0.020 | -1.609 |
| Bivariate Pearson Chi-Square | |  |  | 4.880 |
| Bivariate Log-Likelihood Chi-square | |  |  | 5.027 |
| Item 4 | Item 9 |  |  |  |
| Category 1 | Category 1 | 0.908 | 0.908 | 0.073 |
| Category 1 | Category 2 | 0.002 | 0.002 | -0.412 |
| Category 2 | Category 1 | 0.088 | 0.088 | -0.075 |
| Category 2 | Category 2 | 0.002 | 0.002 | 0.458 |
| Bivariate Pearson Chi-Square | |  |  | 0.384 |
| Bivariate Log-Likelihood Chi-square | |  |  | 0.381 |
| Item 5 | Item 6 |  |  |  |
| Category 1 | Category 1 | 0.911 | 0.908 | 0.601 |
| Category 1 | Category 2 | 0.047 | 0.051 | -0.787 |
| Category 2 | Category 1 | 0.025 | 0.029 | -1.034 |
| Category 2 | Category 2 | 0.016 | 0.013 | 1.532 |
| Bivariate Pearson Chi-Square | |  |  | 3.976 |
| Bivariate Log-Likelihood Chi-square | |  |  | 3.842 |
| Item 5 | Item 7 |  |  |  |
| Category 1 | Category 1 | 0.944 | 0.941 | 0.505 |
| Category 1 | Category 2 | 0.015 | 0.018 | -0.898 |
| Category 2 | Category 1 | 0.033 | 0.036 | -0.639 |
| Category 2 | Category 2 | 0.008 | 0.005 | 1.606 |
| Bivariate Pearson Chi-Square | |  |  | 3.767 |
| Bivariate Log-Likelihood Chi-square | |  |  | 3.490 |
| Item 5 | Item 8 |  |  |  |
| Category 1 | Category 1 | 0.917 | 0.918 | -0.066 |
| Category 1 | Category 2 | 0.041 | 0.041 | 0.096 |
| Category 2 | Category 1 | 0.031 | 0.031 | 0.111 |
| Category 2 | Category 2 | 0.010 | 0.011 | -0.194 |
| Bivariate Pearson Chi-Square | |  |  | 0.058 |
| Bivariate Log-Likelihood Chi-square | |  |  | 0.059 |
| Item 5 | Item 9 |  |  |  |
| Category 1 | Category 1 | 0.956 | 0.956 | 0.175 |
| Category 1 | Category 2 | 0.002 | 0.003 | -0.632 |
| Category 2 | Category 1 | 0.039 | 0.040 | -0.185 |
| Category 2 | Category 2 | 0.002 | 0.001 | 1.065 |
| Bivariate Pearson Chi-Square | |  |  | 1.564 |
| Bivariate Log-Likelihood Chi-square | |  |  | 1.405 |
| Item 6 | Item 7 |  |  |  |
| Category 1 | Category 1 | 0.923 | 0.921 | 0.394 |
| Category 1 | Category 2 | 0.013 | 0.015 | -0.850 |
| Category 2 | Category 1 | 0.054 | 0.056 | -0.462 |
| Category 2 | Category 2 | 0.010 | 0.008 | 1.206 |
| Bivariate Pearson Chi-Square | |  |  | 2.369 |
| Bivariate Log-Likelihood Chi-square | |  |  | 2.284 |
| Item 6 | Item 8 |  |  |  |
| Category 1 | Category 1 | 0.894 | 0.900 | -0.832 |
| Category 1 | Category 2 | 0.042 | 0.036 | 1.345 |
| Category 2 | Category 1 | 0.054 | 0.049 | 1.167 |
| Category 2 | Category 2 | 0.010 | 0.015 | -2.057 |
| Bivariate Pearson Chi-Square | |  |  | 7.274 |
| Bivariate Log-Likelihood Chi-square | |  |  | 7.757 |
| Item 6 | Item 9 |  |  |  |
| Category 1 | Category 1 | 0.933 | 0.933 | -0.102 |
| Category 1 | Category 2 | 0.003 | 0.003 | 0.525 |
| Category 2 | Category 1 | 0.063 | 0.062 | 0.104 |
| Category 2 | Category 2 | 0.001 | 0.002 | -0.691 |
| Bivariate Pearson Chi-Square | |  |  | 0.763 |
| Bivariate Log-Likelihood Chi-square | |  |  | 0.821 |
| Item 7 | Item 8 |  |  |  |
| Category 1 | Category 1 | 0.931 | 0.932 | -0.234 |
| Category 1 | Category 2 | 0.046 | 0.045 | 0.284 |
| Category 2 | Category 1 | 0.018 | 0.017 | 0.471 |
| Category 2 | Category 2 | 0.005 | 0.006 | -0.749 |
| Bivariate Pearson Chi-Square | |  |  | 0.856 |
| Bivariate Log-Likelihood Chi-square | |  |  | 0.892 |
| Item 7 | Item 9 |  |  |  |
| Category 1 | Category 1 | 0.974 | 0.973 | 0.203 |
| Category 1 | Category 2 | 0.003 | 0.004 | -0.550 |
| Category 2 | Category 1 | 0.022 | 0.022 | -0.223 |
| Category 2 | Category 2 | 0.001 | 0.001 | 1.244 |
| Bivariate Pearson Chi-Square | |  |  | 1.899 |
| Bivariate Log-Likelihood Chi-square | |  |  | 1.564 |
| Item 8 | Item 9 |  |  |  |
| Category 1 | Category 1 | 0.946 | 0.945 | 0.119 |
| Category 1 | Category 2 | 0.002 | 0.003 | -0.482 |
| Category 2 | Category 1 | 0.050 | 0.050 | -0.125 |
| Category 2 | Category 2 | 0.002 | 0.001 | 0.725 |
| Bivariate Pearson Chi-Square | |  |  | 0.772 |
| Bivariate Log-Likelihood Chi-square | |  |  | 0.726 |
|  |  |  |  |  |
| **Overall Bivariate Pearson Chi-Square** | |  |  | 78.873 |
| **Overall Bivariate Log-Likelihood Chi-square** | |  |  | 78.58 |
